# Schlangenbiss der Hakennasennatter (*Heterodon nasicus*)

**DOI:** 10.1007/s00105-021-04923-0

**Published:** 2021-12-03

**Authors:** Ulrike Nikfarjam, Stephan Grabbe, Florian Butsch

**Affiliations:** Hautklinik der Universitätsmedizin Mainz, Langenbeckstr. 1, 55131 Mainz, Deutschland

**Keywords:** Hakennasennatter, *Heterodon nasicus*, Schlangenbiss, Lokalreaktion, Therapie, Western hognose snake, *Heterodon nasicus*, Snake bite, Local reaction, Therapy

## Abstract

Es gibt bisher wenige beschriebene Fälle von Bissen durch die Hakennasennatter (*Heterodon nasicus*), die meist zu einer Lokalreaktion führten. Wir berichten über eine ausgeprägte Lokalreaktion nach dem Biss einer als Haustier gehaltenen Hakennasennatter (*Heterodon nasicus*). Eine antiseptische Lokaltherapie und Antibiose zur Infektionsprophylaxe sind zu empfehlen, ebenfalls Laborkontrollen zum Ausschluss einer systemischen Beteiligung. An die Überprüfung des Tetanusschutzes sollte gedacht werden.

## Abstract (en)

There are few reports of snake bites by western hognose snakes (*Heterodon nasicus*), most of which have been associated with local reactions. We report a patient with pronounced local swelling after a bite by a western hognose snake. Local antiseptic therapy and systemic antibiotics are recommended for infection prophylaxis; in addition, laboratory tests should be run to exclude systemic involvement. Regarding tetanus vaccination status, a booster should be considered according to current guideline recommendation.

## Falldarstellung

### Anamnese

Wir berichten über eine 24-jährige Patientin, die sich als Notfall in unserer Poliklinik vorstellte. Die Patientin gab an, etwa 2 Stunden zuvor während der Fütterung von ihrer Hakennasennatter (*Heterodon nasicus*) am linken Daumen gebissen worden zu sein. Die Patientin hielt die Schlange erst seit 2 Tagen als Haustier. Die Schlange habe sich für etwa 10 Minuten festgebissen, bevor es der Patientin gelungen sei, sie zu entfernen. Innerhalb von Minuten sei es zu einer deutlichen Schwellung gekommen. Sie habe mit Wasser gespült und den Daumen gekühlt. Bis auf eine leichte Dyspnoe, welche die Patientin auf die Aufregung zurückführte, war sie ansonsten beschwerdefrei. Keine chronischen Erkrankungen. Keine Dauermedikation. Keine Allergien oder Unverträglichkeiten.

### Klinischer Befund

**Figure 1:**
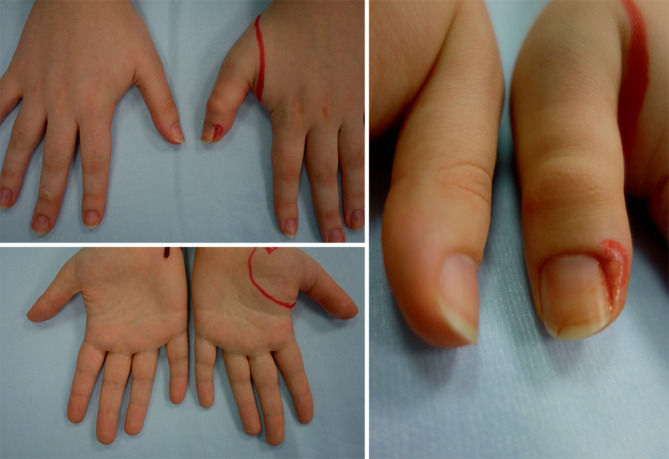

Klinisch zeigte sich im Seitenvergleich eine deutliche Schwellung des linken Daumens. Kleine Erosion der Nagelfalz am Ort des Bisses (Abb. 1).

## Diagnose

Schlangenbiss durch eine Hakennasennatter (*Heterodon nasicus*).

## Therapie und Verlauf

Die hiesige Giftnotrufzentrale wurde umgehend konsultiert. Ein spezifisches Antidot für das Gift der Hakennasennatter (*Heterodon nasicus*) steht nicht zur Verfügung*.* Eine systemische Giftwirkung ist jedoch auch nicht zu erwarten. Klinische Überwachung und Gerinnungskontrollen wurden angeraten, ebenso eine symptomatische Therapie der Lokalreaktion sowie eine antiseptische und ggf. antibiotische Therapie.

Wir behandelten lokal antiseptisch mit Octenidin über mehrere Tage und gaben aufgrund der Schwellung 100 mg Methylprednisolon und eine Ampulle Dimetindenmaleat als Kurzinfusion. Nach Überwachung der Patientin für 6 h zeigte sie sich klinisch stabil. Die erneut kontrollierten Gerinnungswerte waren unauffällig. Wir verschrieben Amoxicillin/Clavulansäure 875 mg/125 mg 2‑mal täglich über 5 Tage und empfahlen eine zeitnahe Überprüfung des Tetanusschutzes.

## Diskussion

**Figure 2:**
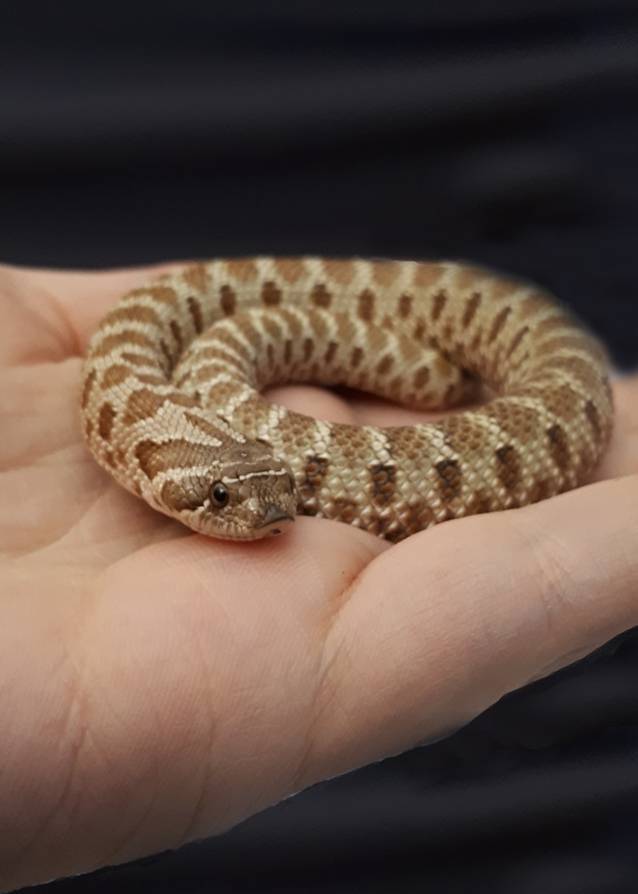

Die *westliche Hakennasennatter* (*Heterodon nasicus*) (Abb. 2) gehört zur Familie der Nattern (*Colubridae*)*.* Sie ist in Mexico und Nordamerika beheimatet und erreicht eine Größe von bis zu 90 cm sowie ein Alter von 15 bis 20 Jahren. Die Ernährung der Tiere besteht aus kleinen Insekten, Mäusen und Kröten [3]. Die Tiere besitzen opistoglyphe, d. h. im hinteren Teil des Oberkiefers liegende, Giftzähne.

Die überwiegende Zahl aller Nattern weltweit ist für den Menschen ungiftig. Ihr Gift ist zu schwach, um Menschen wesentlich schaden zu können.

Ebenso wie die *Ringelnatter* (*Natrix natrix*) hat auch die Hakennasennatter nur kleine, hinten im Schlangenmaul liegende, gefurchte Giftzähne. Die *Giftnattern* (*Elapidae*) hingegen weisen lange gefurchte Zähne im vorderen Teil des Mauls auf. Um überhaupt eine wirksame Menge Gift zu applizieren, muss eine Hakennasennatter ihre Beute deshalb besonders lang im Maul festhalten und mit kauenden Bewegungen das Gift einmassieren. Das erklärt auch in der Kasuistik die relativ geringe Reaktion im Gewebe des Daumens nach dem immerhin 10-minütigen Beißakt. Nattern, die eine für den Menschen spürbare Menge Gift applizieren, aber ihre kleinen Giftzähne versteckt im hinteren Teil des Mauls tragen und sich für den Betrachter damit auf den ersten Blick als „harmlose Nattern“ darstellen, werden auch als *„Trugnattern“* bezeichnet.

Die Hakennasennatter (*Heterodon nasicus*) kennt keinen Verteidigungsbiss, Bissunfälle sind zumeist auf Fütterungsversuche zurückzuführen [8]. Besonders häufig passiert dies, wenn lebende Tiere, beispielsweise Mäuse, als Futter gereicht werden und der Schlangenhalter keinen dicken Handschuh trägt. Die Schlange erkennt nur, dass ihr ein warmblütiges Beutetier zugeführt wird, und kann nicht zwischen Beutetier und der Hand des Schlangenhalters unterscheiden.

Es gibt keine offiziellen Zahlen, wie häufig die Hakennasennatter (*Heterodon nasicus*) in Deutschland oder Europa als Haustier gehalten wird. In einigen deutschen Bundesländern (Schleswig-Holstein, Bayern u. a.) ist die Haltung der Hakennasennatter verboten. Weitere Schlangen neben der Hakennasennatter (*Heterodon nasicus*), die als Haustier gehalten werden, sind z. B. die harmlose Kornnatter (*Pantherophis guttatus*), aber auch Riesen- und Giftschlangen. Schätzungen aus der Halterszene zufolge werden in Deutschland ca. 250.000 Riesen- und 100.000 Giftschlangen gehalten. Deutschland bietet 7 Schlangenarten ihren natürlichen Lebensraum, von denen lediglich 2 giftig sind (vgl. Tab. 1). Schätzungen gehen von 15.000 bis 20.000 Schlangenbissen im Jahr in Europa durch in der Natur vorkommende Schlangen aus [7].

**Table 1:** 

Schlangenart	Beschriebene Reaktionen nach Biss	Besonderheit	Behandlung
Kreuzotter (*Vipera berus*)	Schmerzhafte Schwellung Erbrechen Atemnot Hypotonie	Beißt nur, wenn sie gereizt wird	Bissstelle hochlagern, ruhigstellen. In schweren Fällen Behandlung mit Antivenin
Aspisviper (*Vipera aspis*)	Lokale Gewebeschäden Generalisierte Ödeme Hypotonie GIT-Symptome Hämolyse Nierenfunktionsstörungen Urtikaria Lokalisierte Angioödeme Asthma Herzbeschwerden	Südlicher Schwarzwald, vom Aussterben bedroht	Bissstelle hochlagern, ruhigstellen. In schweren Fällen Behandlung mit Antivenin
Äskulapnatter (*Zamenis longissimus*)	Ungiftig	Längste Schlange Deutschlands	–
Barrenringelnatter (*Natrix helvetica*)	Ungiftig	Vorkommen in Westdeutschland	–
Ringelnatter (*Natrix natrix*)	Ungiftig	Häufigste Schlange in Deutschland	–
Würfelnatter (*Natrix tessellata*)	Ungiftig	Seltenste Schlange in Deutschland, heimisch in Rheinland-Pfalz	–
Schlingnatter (*Coronella austriaca*)	Ungiftig	Umschlingt und erstickt ihre Beute	–

Es gibt wenige beschriebene Fälle über den Biss einer Hakennasennatter (*Heterodon nasicus*). Es gibt keine verlässlichen Angaben über die Häufigkeit von Bissen der Hakennasennatter (*Heterodon nasicus*). Klinisch wurden Ödeme unterschiedlichen Schweregrades, unkomplizierte Risswunden und lokale Schmerzhaftigkeit beschrieben. Die Stärke der Reaktion korrelierte in der Mehrzahl der Fälle mit der Dauer des Bisses. Unklar ist bisher, ob die Klinik durch die toxische Wirkung des Duvernoy-Sekrets oder auf eine Typ-I-Hypersensitivität oder eine Kombination von beidem zurückzuführen ist [9]. Im Jahr 2000 untersuchten Hill und Mackessy die Speichelsekrete von 12 Schlangenarten und stellten hierbei fest, dass in den Speichelsekreten der Hakennasennatter (*Heterodon nasicus*) geringe Giftmengen nachweisbar sind und keine nachweisbaren thrombin-, hyaluronidase- oder kallikreinartigen Aktivitäten festgestellt werden konnten [4].

Eine Typ-I-Überempfindlichkeit gegen Schlangengifte ist eine bekannte Folge der Sensibilisierung durch früheren Kontakt mit Schlangengiften. Zudem kann eine Atopieneigung ein prädisponierender Faktor für eine Reaktion sein [2]. Wenn begleitend eine Wespen- oder Bienengiftallergie vorliegt, kann eine stärkere Reaktion auftreten. Die Ursachen hierfür sind noch nicht abschließend geklärt, es wird eine allgemein erhöhte Bereitschaft zur Histaminfreisetzung und eine Kreuzreaktion durch IgE-Bindung an homologe Proteinallergene diskutiert [5]. Die Frage nach einer Typ-I-Reaktion als Ursache kann durch die Beurteilung der Serum-IgE-Spiegel (in bestimmten Laboren mit Vorlage einer Allergenprobe, d. h. einem Aliquot vom Schlangengift, durchführbar) und des klinischen Ansprechens auf Kortikosteroidbehandlungen beantwortet werden [6].

Neben den beschriebenen lokalen Effekten wurde im Jahr 2019 erstmalig eine systemische Reaktion auf einen Hakennasennattern-Biss beschrieben*. *Eine Thrombozytopenie resultierte aus einem Biss, welche sich im Verlauf von 4 Monaten nach dem Biss unter symptomatischer Therapie stabilisierte [1]. Dies legt nahe, nach einem Schlangenbiss die Gerinnungswerte und das Blutbild zu kontrollieren.

## Fazit für die Praxis

Schlangenbisse sind selten, treten aber dennoch gelegentlich im klinischen Alltag auf. Nach ausführlicher Anamnese sollte eine Rücksprache mit der Giftnotrufzentrale erfolgen. Es empfehlen sich die Kontrolle des Tetanusstatus sowie eine Laborkontrolle zur Detektion des Gerinnungsstatus und des Blutbildes. Zur Vermeidung einer Superinfektion kann mit einem β‑Laktam-Antibiotikum behandelt werden. Eine spezifische Behandlung nach Schlangenbiss der Hakennasennatter (*Heterodon nasicus*) existiert nicht, klinische und laborchemische Verlaufskontrollen sollten erfolgen. Eine Bestimmung des spezifischen IgE kann helfen, das Risiko schwerer Reaktionen bei zukünftigen Bissereignissen abzuschätzen.
